# Peroxynitrite-mediated inactivation of heme oxygenases

**DOI:** 10.1186/1471-2210-4-26

**Published:** 2004-10-21

**Authors:** Robert Kinobe, Yanbin Ji, Kanji Nakatsu

**Affiliations:** 1 Department of Pharmacology and Toxicology, Faculty of Health Sciences, Queen's University, Kingston, Ontario, K7L 3N6, CANADA

## Abstract

**Background:**

Endogenous nitric oxide (NO) and carbon monoxide (CO) are generated by nitric oxide synthase and heme oxygenase, respectively. Like NO, CO has been accepted as an important cellular signaling molecule in biological systems. An up-regulation in both gene and protein expression of heme oxygenase-1 (HO-1) under oxidative/nitrosative stress has been well documented, and the protective role of HO-1 and HO-2 against oxidative damage is proposed. However, data on the direct effect of reactive oxygen/nitrogen species (ROS/RNS) on HO function is incomplete. Using gas chromatography to quantify carbon monoxide (CO) formation from heme oxidation, we investigated the effects of peroxynitrite (ONOO^-^) on the *in vitro *catalytic activity of rat spleen (HO-1) and brain (HO-2) microsomal heme oxygenases.

**Results:**

Exposure to ONOO^- ^led to concentration-dependent but reversible decreases in the activity of microsomal rat spleen and brain HO activity. Spleen HO activity was 100-fold more sensitive to ONOO^-^-dependent inactivation compared to that of the brain, with IC_50 _values of 0.015 ± 0.005 mM and 1.25 ± 0.25 mM respectively. Inhibition of both rat spleen and brain microsomal HO activity was also observed with tetra-nitromethane, a tyrosine nitrating agent, as well as two NO donors, S-nitrosoglutathione (GSNO) and diethylamine NONOate (DEA-NONOate). However, no additive effect was found following the application of NO donors and ONOO^- ^together.

**Conclusion:**

These results indicate that ONOO^- ^may regulate HO-1 and HO-2 activities by mechanisms that involve different interactions with these proteins. It is suggested that while nitration of tyrosine residues and oxidation of sulfhydryl groups may be involved, consideration should be given to other facets of ONOO^- ^chemistry. This inhibition of HO activity offers a mechanism for cross talk between the nitric oxide synthase and HO systems.

## Background

Heme oxygenases (HO, EC 1.14.99.3) are a highly conserved family of proteins that catalyse the oxidative cleavage of heme at the α-meso carbon to yield equimolar amounts of iron, carbon monoxide (CO) and biliverdin. Biliverdin is subsequently reduced to bilirubin by biliverdin reductase. Three distinct isozymes of HO (HO-1, HO-2 and HO-3) have been identified. HO-1 (the inducible isoform) is predominantly expressed in the spleen, the primary site of heme catabolism but has been detected in many different tissues including the liver and the kidney. Several substances and conditions may induce the expression of HO-1. The involvement of HO and products of heme catabolism have been studied extensively with respect to oxidative stress, ischemia, hypoxia and protection against transplant rejection [1-4]. HO-2 (the constitutive isoform) is predominantly expressed in the testes and the brain where HO-2 dependent CO production is thought to aid neuronal function [5-7]. HO-3 is also a constitutive isoform, which shares 90% homology with HO-2 but has very limited catalytic function [8].

Increasing amounts of evidence suggest that products of heme catabolism have cytoprotective roles such as anti-inflammation, anti-apoptosis and anti-proliferation. Biliverdin and bilirubin have anti-oxidant and anti-inflammatory properties [9-11], while iron is known to regulate transferrin, ferritin and nitric oxide synthase gene expression [12,13]. An up regulation in the synthesis of transferrin and ferritin enhances the binding, transport and storage of iron thus serving as an important control mechanism against the oxidative effects of iron. CO, like NO, has been accepted as an important cellular signalling molecule in biological systems. For example, both NO and CO are known to activate soluble guanylyl cyclase, resulting in elevated cGMP and the cGMP-mediated dilatation of blood vessels. CO also mediates vasodilation by directly activating the calcium-dependent potassium channels in vascular smooth muscle cells [14,15]. In addition, CO inhibits platelet aggregation and proliferation of vascular smooth muscle cells, inhibits apoptosis, and stimulates angiogenesis [16-19]. Because of the diversity in the effects of heme catabolism, several studies have suggested that induction of HO-1 expression by oxidising agents may serve as a defense mechanism against oxidative stress *in vivo*. For example, an induction in the expression of HO-1 prevents superoxide associated endothelial cell sloughing in diabetic rats [20]. Similarly, it has been demonstrated that ONOO^-^, a potent oxidizing agent generated by the interaction of NO and superoxide radical [21], causes a concentration-dependent increase in HO-1 protein expression and enzyme activity in rat aortic endothelial cells [22-24], as well as human colorectal adenocarcinoma cells [25]. These studies indicate that ONOO^- ^regulates the expression of HO-1 and that the heme oxygenase pathway contributes to protection against the cytotoxic effects of ONOO^- ^which are due to its reactivity with cellular macromolecules such as the covalent modification of tyrosine, cysteine, methionine or tryptophan residues, oxidation of nucleic bases or the scavenging of cellular antioxidants such ascorbate and urate [26]. It is proposed that the ONOO^-^-mediated HO-1 induction might occur via an interactive signalling mechanism that modulates oxidative stress responses but direct effects of ONOO^- ^on HO activity have not been studied. In this work, we examined the effect of ONOO^- ^on HO catalytic activity in rat spleen and brain microsomes respectively.

## Results

### Effect of ONOO^- ^and TNM on Microsomal HO activity

**Figure 1:**
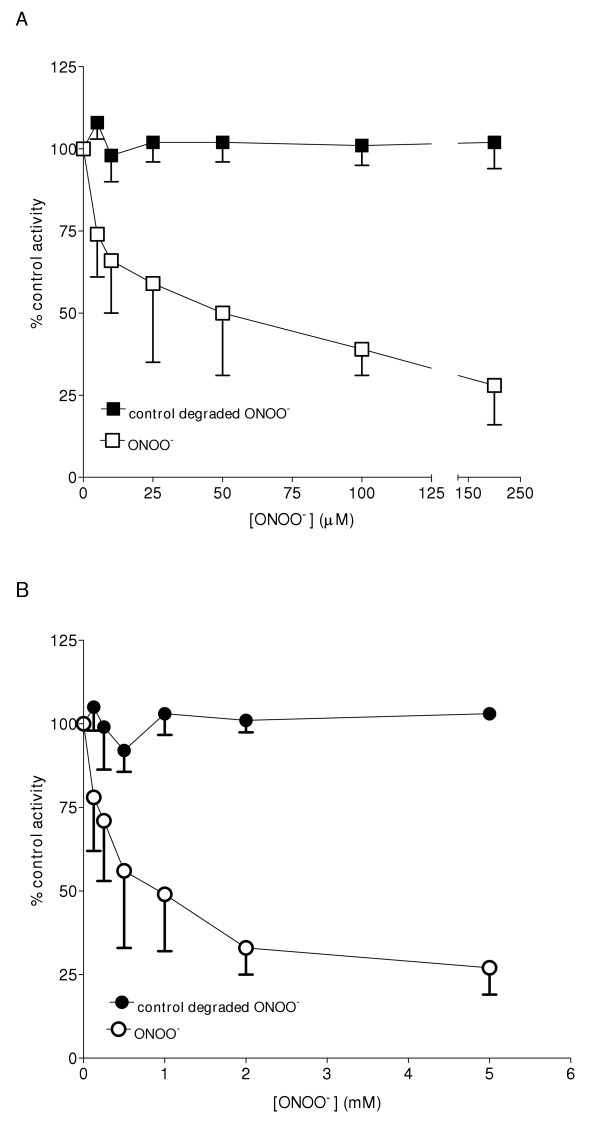
**ONOO^-^-mediated inactivation of HO-1 and HO-2. **Rat spleen (Figure 1A) and brain microsomes (Figure 1B) (50–100 μg protein) were treated with indicated concentrations of ONOO^- ^or degraded ONOO^- ^in 100 mM potassium phosphate, pH 7.4 at room temperature for 10 seconds. The reaction was stopped by dilution of the reaction mixture to a protein concentration of (0.5 mg/mL) spleen microsomes and (1 mg/mL) brain microsomes in 100 mM phosphate buffer containing 1 mM NADPH and 50 μM methemalbumin. Incubations of the pretreated microsomal fractions were performed for 15 min and enzyme activity was determined by the quantitation of CO formed in the reaction mixture. Data are presented as the mean ± SD of triplicate experiments. The rates of CO formation in the control reactions were 12 ± 1 and 5 ± 1 pmoles CO/min/mg protein for spleen and brain microsomes respectively. IC_50 _values for HO-1 and HO-2 were 0.015 ± 0.005 mM and 1.25 ± 0.25 mM respectively.

**Figure 2:**
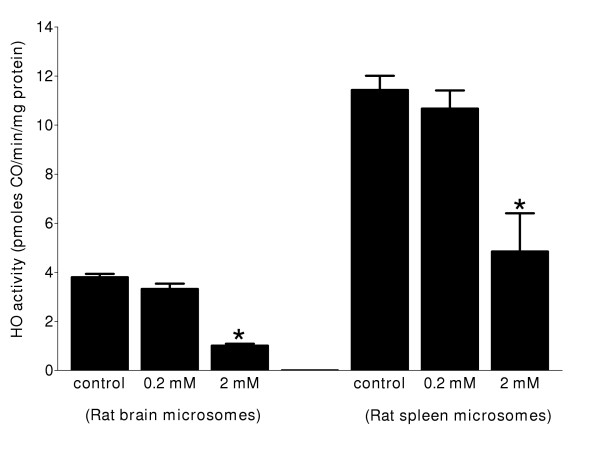
**Effect of TNM-mediated nitration on the catalytic activity of HO. **HO-1 (rat spleen microsomes and HO-2 (rat brain microsomes). Microsomal protein (50–100 μg) was incubated with 0.2 mM or 2 mM (final concentration) of TNM in 100 mM potassium phosphate buffer, pH 7.4, at 37°C for 20 minutes. The reaction was stopped by dilution of the reaction mixture to a protein concentration of (0.5 mg/mL) in 100 mM phosphate buffer containing 1 mM NADPH and 50 μM methemalbumin. Incubations of the pretreated microsomal protein were done for 15 min and enzyme activity was determined by the quantitation of CO formed in the reaction mixture. Data are presented as the mean ± SD of triplicate experiments. The rate of CO formation in control reactions was 11.4 ± 0.6 and 4.0 ± 0.14 pmoles CO/min/mg protein for HO-1 and HO-2 respectively. The asterisk denotes significant inhibition of the respective HO activity using a one-way ANOVA, P ≤ 0.05.

Exposure of either rat spleen or brain microsomes to ONOO^- ^resulted in decreases of HO activity in a concentration dependent manner (Figure 1A and Figure 1B). HO-1 (spleen microsomes) was more sensitive towards ONOO^-^-mediated inactivation compared to HO-2 (brain microsomes) (Figure 1A and Figure 1B). The IC_50 _values for inhibition of HO activity in rat spleen and brain microsomes were 0.015 ± 0.005 mM and 1.25 ± 0.25 mM, respectively. The same concentration of degraded ONOO^- ^did not show any effects on HO activity in rat spleen or brain microsomal fractions. Maximal inhibition for microsomal HO-1 and HO-2 activity was approximately 70%, beyond which there was no further inhibitory effect with increasing concentrations of ONOO^-^. To investigate whether effects of ONOO^- ^were due to nitration of tyrosine residues, spleen and brain microsomal fractions were treated with TNM, a known tyrosine-nitrating agent. It is shown that HO activity in rat spleen as well as brain microsomal fractions was significantly reduced by TNM albeit at higher concentrations compared to ONOO^- ^(Figure 2).

### Effects of sulfhydryl modifying reagents on HO activity

Since ONOO^- ^may affect protein targets by the oxidation of sulfhydryl groups as well as the nitration of tyrosine residues, we examined and compared the effect of other sulfhydryl modifying reagents on HO activity. Rat spleen or brain microsomal protein was treated with different sulfhydryl modifying reagents such as GSNO, DEA-NONOate, NEM and H_2_O_2_, and a combination of equimolar concentrations of ONOO^- ^and GSNO or ONOO^- ^and DEA-NONOate. We found that GSNO caused a concentration dependent inactivation of both HO-1 and HO-2. Consistent with the effect of ONOO^-^, GSNO was more active against HO-1 than HO-2 (Table 1). In contrast, DEA-NONOate and NEM caused a significant decrease in the catalytic activity of HO-1 at the two concentrations tested (0.2 mM and 2 mM), but did not inhibit HO-2 (Table 1). Pre-treatment with equimolar concentrations of ONOO^- ^and DEA-NONOate or GSNO did not result in further inactivation of HO-1 or HO-2 than ONOO^- ^alone. H_2_O_2 _at concentrations as high as 2 mM had no significant effect on the catalytic activity of either isozymes.

**Table 1:** Effect of NO donors and sulfhydryl modifying reagents on the catalytic activity of HO-1 (spleen microsomes) and HO-2 (brain microsomes)

	Heme oxygenase activity (pmoles CO/min/mg protein)	
Experimental conditions	HO-1 (Spleen microsomes)	HO-2 (Brain microsomes)

Control	11.9 ± 0.6	3.1 ± 0.5
ONOO^-^		
0.2 mM	5.4 ± 0.9*	2.7 ± 0.6
2 mM	3.8 ± 0.9*	1.4 ± 0.3*
GSNO		
0.2 mM	6.5 ± 0.3*	2.9 ± 0.5
2 mM	4.2 ± 0.2*	1.4 ± 0.1*
DEA-NONOate		
0.2 mM	4.7 ± 0.4*	2.9 ± 0.7
2 mM	3.6 ± 0.4*	2.4 ± 0.5
NEM		
0.2 mM	8.7 ± 0.8	2.9 ± 0.3
2 mM	3.4 ± 0.5*	2.6 ± 0.4
H_2_O_2_		
0.2 mM	11.3 ± 0.2	3.0 ± 0.4
2 mM	11.8 ± 1.5	3.2 ± 0.6
ONOO^- ^and GSNO		
0.2 mM	4.3 ± 0.6*	2.8 ± 0.4
2 mM	4.0 ± 0.2*	1.3 ± 0.2*
(ONOO^- ^and DEA-NONOate)		
0.2 mM	4.7 ± 0.1*	2.6 ± 0.2
2 mM	3.9 ± 0.6*	1.4 ± 0.3*

Microsomal protein (50–100 μg) was incubated with 0.2 mM or 2 mM (final concentration) of ONOO^-^, GSNO, DEA-NONOate, NEM, H_2_O_2_, equimolar concentrations of ONOO^- ^and GSNO or ONOO^- ^and DEA-NONOate in 100 mM potassium phosphate buffer, pH 7.4, at 37°C for 20 minutes. The reaction was stopped by dilution of the reaction mixture to a protein concentration of (0.5 mg/mL) in 100 mM phosphate buffer containing 1 mM NADPH and 50 μM methemalbumin. Incubations of the pretreated microsomal fractions were done for 15 min and enzyme activity was determined by the quantitation of CO formed in the reaction mixture. Data are presented as the mean ± SD of triplicate experiments. Asterisks denote significant inhibition of the respective HO activity using a one-way ANOVA, P ≤ 0.05.

To test whether effects of ONOO^- ^and the sulfhydryl modifying reagents on HO-1 and HO-2 are reversible, ONOO^- ^and DEA-NONOate were pre-incubated with rat spleen and brain microsomal protein for 20, 60 and 120 minutes prior to evaluation of HO activity. The inactivation of HO-1 by ONOO^- ^and DEA-NONOate and HO-2 by ONOO^- ^was reversible following prolonged pre-incubation time (60–120 minutes) Table 2. However, total HO activity in brain and spleen microsomal protein was not affected by the prolonged pre-incubation for 60–120 minutes in the absence of ONOO^- ^and DEA-NONOate.

**Table 2:** Time dependent reversibility of the effect of ONOO^- ^and DEA-NONOate on the catalytic activity of HO-1 (spleen microsomes) and HO-2 (brain microsomes)

		**Heme oxygenase activity (pmoles CO/min/mg protein) at different pre-incubation times**		
**Experimental conditions**		**20 min**	**60 min**	**120 min**

**Spleen microsomes (HO-1)**	ONOO^-^			
	Control	10.4 ± 1.0	10.8 ± 0.8	8.7 ± 0.9
	0.2 mM	3.6 ± 1.4*	10.5 ± 0.8	8.3 ± 1.1
	DEA-NONOate			
	Control	10.6 ± 2.0	11.5 ± 1.3	9.4 ± 0.2
	0.2 mM	6.6 ± 0.7*	11.1 ± 1.4	8.9 ± 0.3
**Brain microsomes (HO-2)**	ONOO^-^			
	Control	3.6 ± 0.5	4.2 ± 0.4	4.4 ± 0.3
	2 mM	1.9 ± 0.1*	4.4 ± 0.3	3.8 ± 0.3
	DEA-NONOate			
	Control	3.6 ± 0.3	4.1 ± 0.3	4.0 ± 0.3
	2 mM	3.0 ± 0.3	3.9 ± 0.1	3.6 ± 0.3

Microsomal protein (50–100 μg) was incubated with 0.2 mM or 2 mM (final concentration) of ONOO^- ^and DEA-NONOate in 100 mM potassium phosphate buffer, pH 7.4, at 37°C for 20, 60 and 120 minutes. The reaction was stopped by dilution of the reaction mixture to a protein concentration of (0.5 mg/mL) in 100 mM phosphate buffer containing 1 mM NADPH and 50 μM methemalbumin. Incubations of the pretreated microsomal fractions were done as described in materials and methods. Data are presented as the mean ± SD of triplicate experiments. Asterisks denote significant inhibition of the respective HO activity using a one-way ANOVA, P ≤ 0.05.

## Discussion

Production of ONOO^- ^*in vivo *is a consequence of oxidative and nitrosative stress, and ONOO^-^-mediated tissue injury is thought to be involved in the pathogenesis of many conditions including atherosclerosis, ischaemia/reperfusion, shock, Alzheimer's disease, diabetes and multiple sclerosis [27-30]. Continuous challenge and exposure to oxidative/nitrosative stressors has led to the evolution of numerous defense mechanisms in biological systems and one such mechanism that has been elucidated by many researchers is the HO/CO system [4,9-11]. ONOO^- ^causes a concentration-dependent increase in HO-1 protein expression suggesting that the HO pathway contributes to protection against the cytotoxic effects of ONOO^- ^[22-24]. Most studies in this field, however, have focussed on the induction of HO-1 mRNA and/or protein expression under different conditions of oxidative/nitrosative stress rather than enzyme activity. Considering the cellular toxicity of ONOO^- ^and the inhibitory effect of ONOO^- ^on numerous enzyme systems, we sought to investigate the direct effect of ONOO^- ^on the catalytic activity of two microsomal HO isozymes.

Rat spleen and brain microsomal fractions were used because of the predominant expression of HO-1 in the spleen and HO-2 in the brain. We have shown that ONOO^- ^inhibits the activity of both HO-1 and HO-2 in a concentration-dependent manner (Figure 1). HO-1 was found to be more sensitive to ONOO^- ^treatment compared to HO-2. Lower concentrations of ONOO^- ^(15 μM, final concentration) decreased HO-1 activity by 50% while a much higher concentration (1.25 mM) was required to decrease HO-2 activity by the same magnitude. Generally, HO catalytic function is dependent on an accessory enzyme NADPH-cytochrome P450 reductase (CPR), which serves as a redox partner during the oxidative break down of heme and the conversion of NADPH to NADP^+^. It is possible therefore, that ONOO^- ^may have altered HO activity indirectly by inactivating CPR. The ONOO^- ^dependent inactivation of recombinant CPR in bacterial membranes has been noted [32], but our attempt to supplement rat microsomal HO-1 and HO-2 with recombinant rat CPR in the presence of a detergent did not attenuate the effect of ONOO^- ^on microsomal HO-1 and HO-2 (data not shown). This suggests that the effects of ONOO^- ^on HO activity may be mediated by mechanisms that are independent of the effect of ONOO^- ^on microsomal CPR activity. The differential effect of ONOO^- ^on the catalytic activity of HO-1 and HO-2 may suggest a selective mechanism on the catalytic function of the inducible enzyme. This may have direct implications in biological systems where ONOO^- ^is generated at high concentrations. The ONOO^-^-mediated increase in HO-1 gene and/or protein expression may be a cellular response at the acute phase. Subsequently, total HO activity may then be maintained in certain ranges in order to retain intracellular oxidative responses and the balance of redox states for normal cellular function. This idea is consistent with other studies, which have shown that induction of HO-1 mRNA and protein expression is not always followed by a proportionate increase in catalytic function. For example, by using quantitative RT-PCR it was found that a 3.8-fold increase in sarcoma-induced HO-1 mRNA expression yields only 2.1-fold increase in total HO activity [31]. Overall, the ONOO^-^-mediated regulation of the catalytic function of HO-1 and differences in the sensitivity of HO-1 and HO-2 may be attributed to disparity in the amino acid compositions and the structure of the two proteins.

Mechanistically, the effects of ONOO^- ^on protein function are mainly due to its reactivity and covalent modification of tyrosine and/or cysteine residues [26]. This often leads to impaired protein function with very few exceptions such as the recently observed nitration and stimulation of the enzymatic activity of microsomal glutathione S-transferase (MGST) [33]. To probe mechanisms of ONOO^-^-mediated inhibition of HO activity observed here, we examined the effects of TNM, another tyrosine-nitrating chemical compound on rat spleen and brain microsomal HO activity. Significant inhibition of HO catalytic activity by TNM was observed for spleen as well as brain microsomal fractions albeit at higher concentrations compared to ONOO^- ^(Figure 2). This suggests that both TNM and ONOO^- ^may inactivate HO activity by nitration of tyrosine residue(s) that is/are important for conservation of HO-1 and HO-2 function. This is consistent with data from the analysis of the complete amino acid sequences of rat and human HO-1 [34,35] and HO-2 [36,37]. However, there was no difference in the effects of TNM on HO-1 and HO-2 (Figure 2), suggesting that the differential effects observed with ONOO^- ^could be due to modification of amino acid residues other than tyrosine. Oxidation of cysteine residues as a putative mechanism underlying ONOO^-^-mediated inactivation of HO was investigated. Effects of GSNO, DEA-NONOate, NEM and H_2_O_2_, or a combination of equimolar concentrations of ONOO^- ^and GSNO or DEA-NONOate on HO activity were tested. GSNO showed similar inhibitory effects to ONOO^- ^but a combination of equimolar concentrations of ONOO^- ^and DEA-NONOate or GSNO did not have any additive effect on the ONOO^-^-mediated inactivation of HO-1 or HO-2. In addition, the inactivation of HO-1 by ONOO^- ^and DEA-NONOate and HO-2 by ONOO^- ^were reversible following prolonged pre-incubation time (60–120 minutes) Table 2. This differential effect between HO-1 and HO-2 following exposure to ONOO^-^, GSNO, DEA-NONOate or NEM is suggestive of qualitative as well as quantitative differences in the distribution of target amino acids such as critical tyrosine and/or cysteine residues in the tertiary structures of these proteins. The amino acid sequences of human and rat HO-2 reveals a conserved core of cysteine residues (Cys 264-Pro 265, Cys 281-Pro 282) [38,39], but there are no cysteine residues in the HO-1 amino acid sequence. Despite this fact, results from our experiments show that HO-1 is more sensitive to inactivation by the NO donors GSNO and DEA-NONOate, and NEM (Table 1). We considered the possibility that ONOO^- ^might exert its effects by interaction with the substrate as has been reported for the interaction of NO with heme [40]. This possibility seems unlikely as such a mechanism would be expected to affect the catalytic rate of HO-1 and HO-2 equally. Another consideration is that the chemistry of ONOO^- ^is richer than is usually discussed; in their review Alvarez and Radi [41] describe the interactions of ONOO^- ^with a number of functional groups that are not often mentioned in the consideration of the interactions of ONOO^- ^with various proteins. Thus, ONOO^- ^inhibition of HO activity could be a result of an interaction with amino acids other than cysteine or tyrosine. This possibility of ONOO^- ^interactions with other amino acids may also explain the reversibility of the inhibition of HO activity by ONOO^-^. Furthermore, it is possible that ONOO^- ^inhibition of HO-1 and HO-2 occurs courtesy of different facets of ONOO^- ^chemistry. Differences in the relative potency of DEA-NONOate, NEM, GSNO and ONOO^- ^may also be attributed to differences in the chemical properties and the electrophilic potentials of these reagents. For instance, the nitric oxide radical (NO^•^) generated from DEA-NONOate is less reactive and unstable compared to NO^+ ^produced from GSNO.

Collectively, our data indicate that ONOO^- ^may be important in the cross-talk between HO and NO systems. While tyrosine residues for HO-1 and/or sulfhydryl groups for HO-2 should be considered as potential targets for ONOO^- ^interactions, other amino acids should be studied in the elucidation of mechanism(s) of ONOO^- ^action on HO isozymes.

## Conclusion

This study documented for the first time the ONOO^-^-mediated inactivation of HO-1 and HO-2. The IC_50 _for HO-1 was approximately 80-fold less than that for HO-2. While conserved tyrosine residues (HO-1) and/or sulfhydryl group(s) (HO-2) may play a critical role in maintaining the functional capacity of heme oxygenases, the consideration of other amino acids is suggested. In addition, the higher sensitivity of HO-1 to ONOO^-^-mediated inactivation may indicate dual regulatory mechanisms on the catalytic function of the inducible enzyme under the conditions of oxidative/nitrosative stress.

## Methods

### Materials

Hemin, ethanolamine, bovine serum albumin (BSA), β-NADPH, N-ethylmaleimide (NEM), tetra-nitromethane (TNM) and reduced glutathione (GSH) were purchased from Sigma Chemical Co. (St. Louis Mo.). Diethylamine NONOate (DEA-NONOate) was obtained from Calbiochem Inc. (Darmstadt, Germany). All other chemicals were reagent grade from a variety of commercial sources.

### Preparation of rat spleen and brain microsomes

Adult male Sprague-Dawley rats (250–300 g) were obtained from Charles River Canada Inc. (St-Constant, Que). The animals were cared for in accordance with the principles and guidelines of the Canadian Council on Animal Care. Microsomal fractions were prepared according to previously described procedures [42,43]. Spleen microsomes were used as a source of HO-1 while brain microsomes were used as a source of HO-2.

### Preparation of ONOO^- ^and treatment of microsomal protein

ONOO^- ^was synthesised from acidified nitrite and H_2_O_2 _according to the method of Beckman [44] and microsomal HO-1 (spleen microsomes) and HO-2 (brain microsomes) were treated with ONOO^- ^following the procedures described by Ji and Bennett [33]. Briefly, microsomes (50 –100 μg protein in 100 mM potassium phosphate, pH 7.4) were exposed to ONOO^- ^at room temperature at indicated concentrations for 10 seconds by adding a small volume during vigorous mixing. HO activity was initiated by diluting the reaction mixture to a protein concentration of (0.5–1 mg/ml) in 100 mM phosphate buffer containing substrates (50 μM methemalbumin, and 1 mM NADPH). To control for the potential effect of nitrite and nitrate that may be formed during the incubation of ONOO^-^, ONOO^- ^was allowed to decompose in phosphate buffer prior to incubation with microsomal protein and determination of enzyme activity for some of the reactions.

### Treatment of microsomes with NO donors, TNM and sulfhydryl modifying reagents

GSNO was prepared by reacting equimolar amounts of sodium nitrite and GSH according to the method described by Ji *et al*., [43]. Microsomal protein (50 – 100 μg) was incubated with 0.2–2 mM GSNO, DEA-NONOate, NEM or H_2_O_2 _in 100 mM potassium phosphate buffer, pH 7.4, at 37°C for 20 minutes. The reaction was stopped by dilution of the reaction mixture to a protein concentration of (0.5–1 mg/mL) in 100 mM phosphate buffer containing (50 μM methemalbumin, and 1 mM NADPH).

### *In vitro *Assay for HO activity

HO activity in rat spleen and brain microsomal fractions was determined by the quantitation of CO formed from the oxygen, CPR and NADPH-dependent degradation of methemalbumin as previously described [45,46]. The reaction mixture contained, (0.5–1 mg/mL microsomal protein, 50 μM methemalbumin, and 1 mM NADPH) in 100 mM phosphate buffer. All incubations for the assay of HO activity were performed under the conditions for which the rate of CO formation (pmol CO. mg^-1 ^protein. min^-1^) was linear with respect time and microsomal protein concentration. CO formation was monitored by gas chromatography according to the method described by Vreman and Stevenson [46].

### Statistical analysis

Data are presented as the mean ± SD from triplicate experiments and statistical analyses were performed by one-way ANOVA. P values ≤ 0.05 was considered to be significant.

## Abbreviations

HO, heme oxygenases; CPR, NADPH-cytochrome P450 reductase; CO, carbon monoxide; ONOO^-^, peroxynitrite; GSNO, S-nitrosoglutathione; DEA-NONOate, diethylamine NONOate; NEM, N-ethylmaleimide; TNM, tetra-nitromethane; ROS/RNS, reactive oxygen/nitrogen species; RT-PCR; reverse transcriptase polymerase chain reaction;  H_2_O_2_, hydrogen peroxide.

## Authors' contributions

RK, YJ and KN were involved in the experimental design and data collection. All authors read and approved the final manuscript.
